# Skin disorder management in oral anticancer drugs by collaboration of hospital pharmacists and community pharmacists

**DOI:** 10.1007/s00520-020-05875-2

**Published:** 2020-11-07

**Authors:** Ryuta Urakawa, Sanae Hashimoto, Hideki Hirohata, Katsunori Sakai, Kayo Matsuura, Yumiko Ito, Masahito Tarutani, Kazumi Kubota, Mikiko Ueda, Etsuko Uejima

**Affiliations:** 1 Department of Pharmacy, Osaka University Dental Hospital, 1-8 Yamada-oka, Suita, Osaka 565-0871 Japan; 2 Graduate School and School of Pharmaceutical Sciences, Osaka University, 1-6 Yamada-oka, Suita, Osaka Japan; 3 Department of Pharmacy, Kinki Central Hospital, 3-1 Kurumazuka, Itami, Hyogo Japan; 4 Department of Pharmacy, Kusatsu General Hospital, 1660 Yabase, Kusatsu, Shiga Japan; 5 Department of Nurse, Kinki Central Hospital, 3-1 Kurumazuka, Itami, Hyogo Japan; 6 Department of Dermatology, Kinki Central Hospital, 3-1 Kurumazuka, Itami, Hyogo Japan; 7 Department of Biostatistics, Yokohama City University School of Medicine, 3-9 Fukuura, Kanazawa-ku, Yokohama, Kanagawa Japan

**Keywords:** PBPM, Chemotherapy, Community pharmacy, Skin toxicity

## Abstract

**Background:**

In Japan, the multidisciplinary team approach in cancer chemotherapy has become quite widespread. However, patients treated with oral anticancer drugs in outpatient clinics usually receive short medical examinations from doctors without any intervention of pharmacists. To improve this medical circumstance, we made a skin disorder manual for community pharmacists and evaluated its feasibility.

**Methods:**

Patients who underwent oral skin toxic chemotherapy from May 1, 2017, to October 31, 2017, were enrolled. The severity of skin toxicities was evaluated based on NCI-CTCAE ver4.0. Skin care and skin disorders were assessed by community pharmacists based on the assessment document arranged by the investigator. Numbers of patients who replied to the assessment, numbers of replies, numbers of assessments and instructions for skin care, and numbers of prescription proposals were evaluated to assess the value of intervention of community pharmacists.

**Results:**

Sixty-two patients were enrolled in this study. Community pharmacy responded to 55 patients (88.7%), for a total of 335 replies. The data described in the replies were as follows: 317 assessments of skin disorders (94.6%), 307 assessments of skin care (91.6%), 248 instructions for skin care (74%), and 19 prescription proposals (5.7%).

**Conclusions:**

Community pharmacists have high motivation for prevention and early detection of skin disorders. Although the number of prescription proposals is small, some proposals have contributed to improving side effects. Collaboration of hospital pharmacists and community pharmacists is important for prevention, early detection, and treatment of skin disorders caused by oral anticancer drugs.

## Introduction

Cancer chemotherapy can cause a variety of side effects, which can lead to patient disadvantages such as lower patient quality of life and dose reduction. Therefore, prevention, early detection, and early treatment of side effects are essential. In Japan, side effect management is generally performed by teams consisting of doctors, nurses, and pharmacists. However, patients who are treated with oral anticancer agents without any injection in outpatient clinics are exceptions, so that they are usually followed up only by a short doctor’s examination without the involvement of pharmacists. This is due to the lack of human resources and high costs, which are difficult problems to solve. In many cases, patient care and chemotherapy management are entrusted to doctors and nurses. Pharmacists are often involved only after side effects have occurred or when the side effects become severe and doctors or nurses are forced to consult with pharmacists.

In order to improve these medical circumstances, various efforts are being performed in Japan. Some evidence has been reported previously about clinical benefits associated with pharmacists’ participation in outpatient chemotherapy [1–4]. However, there are few cases and few reports that hospital pharmacists are involved in care of patients who are treated with oral anticancer drugs alone [4, 5]. Even in cases when pharmacists examine such outpatients, it is quite difficult to intervene in all patients because of limited human resources. A tracing report or protocol-based pharmacotherapy management (PBPM) with community pharmacies is another attempt to follow outpatients. PBPM is a derivative of collaborative drug therapy management (CDTM). In Japan, where pharmacists do not have the authority to order prescriptions and tests, pharmacists provide drug treatment in collaboration with physicians and others based on a protocol prepared and agreed upon in advance by physicians and pharmacists, utilizing their pharmaceutical knowledge and skills. However, performance rates and clinical benefits such as survival benefit or risk reduction of these attempts are unknown. In addition, there is no fixed protocol or rule in the method used jointly by the hospital pharmacists and community pharmacists, and there are disparities depending on hospitals and pharmacies.

In this study, working with skin and cancer experts in our hospital, we produced an instruction manual for community pharmacies to manage skin toxicities caused by chemotherapy. The aim of this manual is for community pharmacists to provide effective advice in order to reduce the incidence and the severity of skin disorders. This pharmacy instruction manual assists prevention and treatment of skin disorders by providing skin care instructions, assessments of skin disorders, and suggestions for prescriptions. Here, we hypothesize that use of an instruction manual for skin disorder management written by skin experts and cancer experts for community pharmacists can prevent the onset or worsening of skin disorders. To investigate this hypothesis, we examined feasibility of our manual by analyzing efforts of community pharmacists.

## Material and methods

### Patient selection

The subjects were cancer patients who underwent oral skin toxic chemotherapy at Kinki Central Hospital from May 1, 2017, to October 31, 2017. Oral skin toxic chemotherapy consisted of capecitabine, regorafenib, gefitinib, erlotinib, afatinib, osimertinib, axitinib, sunitinib, sorafenib, everolimus, lapatinib, and pazopanib. To be eligible for the study, all patients were required to be at least 18 years old and to have received an oral anticancer drug at a community pharmacy by out-of-hospital prescription. Cancer type and duration from initial administration were not included in the criteria. The exclusion criteria included patients who constantly received skin disorder management by an in-hospital pharmacist. We received institutional review board approval to start this clinical research. Before enrolling in this study, patients signed the informed consent form after getting sufficient explanation.

### Study objectives

The purpose of this study was to evaluate feasibility of our manual. In order to assess its feasibility, we examine the extent to which community pharmacists adhere to this manual for skin disorder management. Another purpose of this study was to examine its effectiveness. In order to assess its effectiveness, we focused on cases in this study population that could have prevented any worsening of skin disorders.

### Methods of skin disorder management

**Fig. 1:**
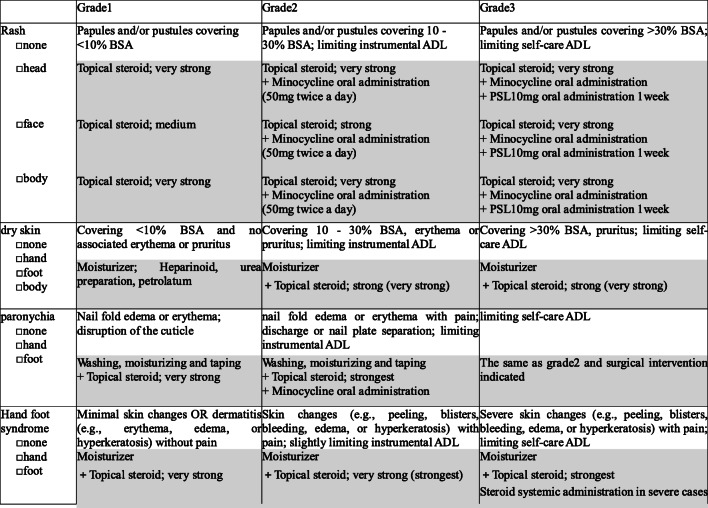
Skin disorders countermeasure document. Topical steroids are divided into five classes based on how strong they are according to guidelines for management of atopic dermatitis 2018 of the Japanese Dermatological Association

**Fig. 2:**
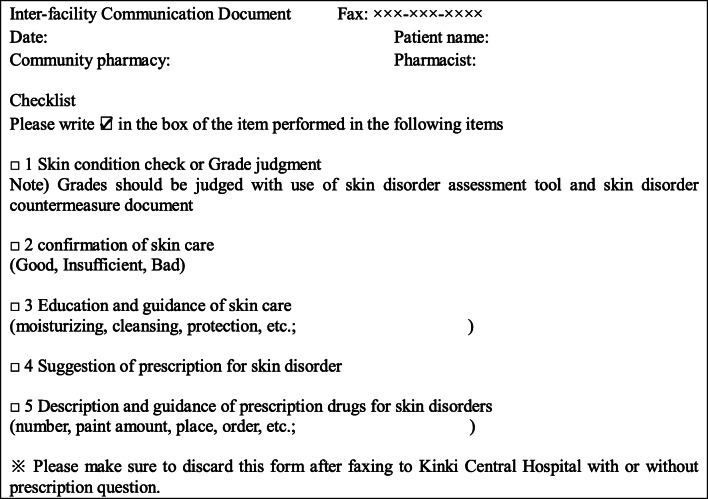
Inter-facility communication document

The author, in cooperation with a dermatologist and a certified nurse in wound, ostomy, and continence nursing, created a skin disorders countermeasure document that can evaluate the severity and measures of skin disorders caused by anticancer drugs (Fig. 1). Additionally, in order to assess the effectiveness of the manual in community pharmacy, we made an inter-facility communication document (Fig. 2).

To ensure knowledge of skin care and measures against skin problems, the author, in cooperation with a dermatologist and a certified nurse in wound, ostomy, and continence nursing—experts who created the countermeasure document—held a joint lecture with community pharmacies of pharmaceutical association in Itami, Japan, where the Kinki Central Hospital is located. Methods of skin disorder management were conveyed to community pharmacies through a lecture, a website, an inter-facility communication document, and a skin disorders countermeasure document. The identification of types of skin disorders and severity evaluation methods focused on those that can be understood by pharmacists from visual inspections and interviews. In addition, the therapies for skin problems recommended by the manual were focused on medications and skin care without any surgical procedures. We explained this study to patients taking oral anticancer drugs at outpatient visits and provided an enrollment document to each patient who agreed to participate. Community pharmacies that received the requesting document followed steps for skin disorder management every time the patient came for medicine. To help prevent skin disorders, community pharmacists evaluated the skin care of patients from the viewpoint of moisturizing, cleansing, and protecting skin, and provided skin care instructions if necessary. If skin disorders developed, the pharmacists assessed their type and severity, assessed whether the required medicines were prescribed and used appropriately, and, if necessary, proposed medication and instructed patient about skin care. Every time the pharmacists performed the skin disorder management, they completed the inter-facility communication document and provided it by fax to pharmacists at Kinki Central Hospital.

### Grading of skin toxicities

Skin toxicities were assessed using the assessment document based on National Cancer Institute Common Terminology Criteria for Adverse Events version 4.0 (NCI-CTCAE v4.0) (Fig. 1). Hand-foot syndrome, rash, paronychia, and dry skin were included in skin toxicities to evaluate. The grade of CTCAE corresponding to the severity of each side effect is described, and the recommended drug and countermeasure are described in the corresponding column. Community pharmacists assessed the severity of skin toxicities and checked prescription medicines for those skin toxicities according to the assessment document.

### Evaluation of research results

In order to understand how much the community pharmacy can perform management based on this manual, the rate of responses was calculated by the ratio of patients who received a reply from the community pharmacy to those who consented to participate. In addition, from the compiled results of the questionnaire, we determined the rates of skin care assessments, skin care instructions, and prescription proposals. We evaluated the contents of the prescription proposals.

## Results

Sixty-seven patients met the eligibility criteria, and 62 patients were enrolled in this study after informed consent. Patient demographic and clinical characteristics are summarized in Table 1. The responses from community pharmacy are summarized in Table 2. We got fax replies for 55 patients (88.7%), for a total of 335 replies from community pharmacies. The items listed in the replies were as follows: 317 assessments of skin disorders (94.6%), 307 assessments of skin care (91.6%), 248 instructions of skin care (74%), 19 prescription proposals (5.7%). The contents of the prescription proposal were as follows: 8 requests for topical steroid preparation, 6 requests for moisturizer, 3 changes in rank for topical steroid preparation, 1 case for change of the base material of topical agent, 1 request for minocycline prescription. Numbers of patients with each grade of skin toxicity are shown in Table 3. Here, we show two cases that were especially successful in controlling their skin disorders with this approach.

**Table 1:** Patient demographic and clinical characteristics

Characteristics	*N* = 62
Age	
Mean (SD)	70.4
Gender	
Male (*n* (%))	31 (50)
Female (*n* (%))	31 (50)
Regimen	
Capecitabine alone	11
Capecitabine plus other chemotherapy agents	27
Gefitinib	8
Elrotinib	7
Others	9

Other chemotherapy agents consisted of cisplatin, oxaliplatin, irinotecan, bevacizumab, trastuzumab, and panitumumab

**Table 2:** Response from community pharmacy

	Enrolled patients	Patient number with fax responses (%)	Total number of fax responses
Total number	62 patients	55 patients (88.7%)	335 cases
① Assessments of skin disorders			317 cases (94.6%)
② Assessments of skin care			307 cases (91.6%)
③ Instructions of skin care			248 cases (74.0%)
④ Prescription proposals			19 cases (5.7%)

**Table 3:** Number of patients with each grade of skin toxicity (NCI-CTCAE ver4.0)

Skin toxicity	Grade 0	Grade 1	Grade 2	Grade 3
Rash	39	6	7	3
Xerosis	20	17	17	1
Paronychia	30	15	9	4
Hand-foot syndrome	24	15	16	0

### Case 1

#### Lung adenocarcinoma, female, erlotinib

The patient was introduced erlotinib under admission control for postoperative recurrence of lung adenocarcinoma. The dosage was reduced from 150 to 50 mg due to diarrhea and fatigue, and treatment was continued during outpatient visit after leaving hospital. Although moisturizers were used to prevent skin disorders, betamethasone valerate was prescribed because of the onset of grade 1 paronychia in the hands and feet. At the community pharmacy, the patient received guidance on taping around the nails to protect, but paronychia was worsened to grade 2. Also, since the patient developed grade 2 acneiform rash and grade 2 dry skin on the whole body, oral minocycline and rank up from betamethasone valerate to difluprednate ointment were proposed by a community pharmacist. The pharmacist also instructed use of betamethasone valerate and difluprednate properly according to degree of the skin disorders. After that, skin rash and dry skin remained at grade 2 and the paronychia was relieved to grade 0. The patient needed no extra medical examination due to a skin disorder other than a regular medical examination.

### Case 2

#### Lung adenocarcinoma, female, gefitinib

Patient was under treatment with gefitinib for lung adenocarcinoma. The betamethasone butyrate propionate ointment was prescribed for erythema of hands and grade 2 acneiform rash on the trunk. Because the skin rash on the trunk was extensive, a community pharmacist proposed the change from ointment to lotion for the trunk while continuing the ointment for the hands. Next week, clobetasol propionate sculpt lotion was prescribed for head discomfort. Because no inflammation was seen in the head, a community pharmacist inquired about the need for steroid preparation. As a result, clobetasol propionate sculpt lotion was ranked down to a betamethasone butyrate propionate lotion. After that, the head symptom did not get worse. Acneiform rash on the trunk improved to grade 1 temporarily, but it worsened to grade 2 again, and steroid external preparation was upgraded to clobetasol propionate lotion. After that, rank down to betamethasone butyrate propionate lotion was suggested by a pharmacist because of improvement of rash, resulting in no worsening. There was no need to have further examination by a primary care doctor.

## Discussion

In Japan, team approaches by doctors, nurses, and pharmacists in cancer treatment have evolved over the past two decades. Although there are some facilities where hospital pharmacists follow up outpatients by interviewing and checking laboratory data, it is impossible to follow up all patients who undergo oral anticancer drugs. It is often seen that side effects that are rarely life-threatening, such as skin toxicities of chemotherapy, are left behind. As a result, patients sometimes have to be hospitalized due to serious side effects in daily clinical practice. Therefore, we considered that cooperation with community pharmacies could realize prevention, early detection, and early treatment of side effects. According to the statistics of the number of doctors and pharmacists at the Ministry of Health, Labour and Welfare in 2018, the number of doctors working in hospitals was about 208,000, and the number of pharmacists was about 54,000. On the other hand, the number of community pharmacists is about 180,000, so if we can get their more active cooperation as a health care provider, we can expand the cancer care workforce.

Recently, the collaborations between community pharmacies and hospital pharmacies have been promoting the display of clinical laboratory test values in prescriptions, the inspection rights of electronic medical records, the use of formulary, and the use of tracing documents. Moreover, demonstrations of their effects have been reported, such as the medical economic effect or the case report that inquiry reference has been effective [6, 7]. Benefits of economic and prescription optimization from PBPM in accordance with community pharmacy and hospital pharmacy have also been reported [8]. Though similar efforts have been reported for chemotherapy, there are no reports that indicate their clinical benefits clearly [9–12]. A past research study reported that more than half of community pharmacists rarely feedback patient information to hospitals, and 14.3% community pharmacists rarely performed tasks such as continuous side effect monitoring and patient guidance, including supportive care [13]. They also reported the percentage of community pharmacists who continuously engaged in pharmaceutical care during chemotherapy as follows: 91.7% checking for the occurrence of side effects, 83.3% providing guidance on countermeasures and prevention of side effects, and 12% providing prescription proposals.

In this study, we made an instruction manual for protection, evaluation, early detection, and treatment of skin disorders. These instructions, aimed at community pharmacists, correspond to procedures performed by hospital pharmacists who performed in-patient services. Furthermore, we considered a program to share the manual with community pharmacies and to feedback information to hospitals. In this study, we examined whether this program could be carried out by community pharmacists in a real-world clinical setting. The rate for receiving responses to 88.7% of patients was extremely high with reference to the past research [13]. In addition, the percentages of 94.6% assessments of skin disorders, 91.6% assessments of skin care, and 74% instructions of skin care were comparable to the previous report. It revealed high motivations and high interests of community pharmacists in prevention and early detection of skin disorders. However, the rate of 5.7% prescription proposals was considered to be low. According to our interviews with several community pharmacists, they sometimes gave up their suggestions because they were not confident. Also, many community pharmacists described detailed skin conditions freely in the inter-facility communication document, many of which seemed to require prescribing proposals. We considered that more active interventions were essential to treat skin disorders. The contents of the prescription proposal were all appropriate, such as the proposal of a moisturizer to prevent skin disorders, and the selection of topical steroids according to the severity of skin disorders. Therefore, we could improve knowledge about prevention and treatment of skin disorders by using a variety of methods: a joint lecture, a website, an inter-facility communication document, and a skin disorders countermeasure document. We conclude that hospital pharmacists who are specialized in cancer treatment should facilitate relationships between community pharmacists and medical teams.

In case 1, guidance on taping for paronychia, suggestion of steroid strength according to the severity of each skin disorder, and suggestion of minocycline administration were performed. The taping method had been acquired through prior instruction from a JSPHCS-certified oncology pharmacist and a certified nurse in wound, ostomy, and continence nursing through a study session. In case 2, changes in the base according to the range of skin disorder and suggestion of steroid strength according to the severity were performed. In both cases, in addition to general medication guidance, advanced prescription suggestions and patient guidance were provided by community pharmacists. Also, the two cases could have avoided unnecessary medical examinations by preventing worsening of skin disorders. We think that improving patient’s adherence is a factor that could prevent the worsening of skin disorders. There may be regional differences in the degree of understanding for skin disorders and the degree of intervention in the treatment of cancer patients. Our study shows that development of an instruction manual may solve this problem.

This study has three limitations. The first is selection bias. Because the manual for skin disorder management was conducted at a single hospital and nearby community pharmacies, the number of subjects was limited. Moreover, cancer type and therapeutic agent are biased by the hospital. It is desirable to conduct multicenter clinical research to address this limitation. The second is evaluation methods of this study. Because no statistical analysis is conducted, it is impossible to draw clear conclusions. However, it is true that there were patients who benefited from the therapeutic intervention of the pharmacist at the community pharmacy. Additionally, this report is the first research that indicates the rate of patients that community pharmacists evaluate and take measures against side effects according to the manual. The third is convenience of the manual. To produce this manual, a great deal of work was required of community pharmacists and hospital pharmacists. When exchanging patient information or inquiring about management, it was necessary to fill out and send an inter-facility communication document by fax or contact by phone. It would be much easier if the information could be exchanged on the Internet. Recently, some studies have reported that smartphone applications have been useful in clinical settings [14–16], including cancer treatment [17–21]. If this manual can be used in smartphone applications, it will be easy to use as a generalized one in more facilities.

In conclusion, the hypothesis that a skin disorder control program made by skin experts for community pharmacists can prevent the onset or worsening of skin disorders was proven to be correct. This research revealed for the first time that a skin care manual made by experts for community pharmacists, which is a form of PBPM, was viable with very frequent responses and high assessment rate. Through this research, we found that community pharmacists performed skin disorder management with high interest under the cooperation of the community pharmacists and hospital pharmacists. In addition, it was found that this type of cooperation is useful for prevention, early detection, and early treatment of skin disorders. These findings show the possibility that community pharmacists can be key care providers along with doctors, nurses, and hospital pharmacists, who ensure not only prescriptions but effects or side effects of medications. Additionally, it is also possible that community pharmacists can contribute to improved patient quality of life, management of chemotherapy, and survival if closer cooperation between hospital and community pharmacy is achieved.

As we previously reported, hand-foot syndrome was the strongest factor in decreasing QoL so that multi-kinase inhibitors, EGFR-TKI, and capecitabine require careful prevention, early detection, and daily medical care [22]. In the future, we will conduct a multicenter clinical study in order to verify the contribution of this approach to clinical benefits such as prevention of worsening and prolongation of PFS by targeting patients treated with multi-kinase inhibitors, EGFR-TKI, and capecitabine.

## References

[1] Iihara H, Ishihara M, Matsuura K, Kurahashi S, Takahashi T, Kawaguchi Y, Yoshida K, Itoh Y (2012). Pharmacists contribute to the improved efficiency of medical practices in the outpatient cancer chemotherapy clinic. J Eval Clin Pract..

[2] Fujii H, Iihara H, Ishihara M, Takahashi T, Yoshida K, Itoh Y (2013). Improvement of adherence to guidelines for antiemetic medication enhances emetic control in patients with colorectal cancer receiving chemotherapy of moderate emetic risk. Anticancer Res..

[3] Imamura M, Ogawa D, Takatori T, Yamaguchi M, Takata T, Hada T, Ota Y, Uehara T (2017). A retrospective study of the effects of oncology pharmacist participation in treatment on therapeutic outcomes and medical costs. Biol Pharm Bull..

[4] Suzuki H, Suzuki S, Kamata H, Sugama Y, Demachi K, Ikegawa K, Igarashi T, Yamaguchi M (2019). Impact of pharmacy collaborating services in an outpatient clinic on improving adverse drug reactions in outpatient cancer chemotherapy. J Oncol Pharm Pract..

[5] Aimono Y, Kamoshida T, Sakamoto R, Nemoto M, Saito Y, Aoyama Y, Maruyama T (2015). Initial evaluation of the efficacy and safety of tablets containing trifluridine and tipiracil hydrochloride--safety measures devised by a multidisciplinary team including a pharmaceutical outpatient clinic. Gan To Kagaku Ryoho..

[6] Igarashi T, Kodawara T, Konno A, Kamitani Y, Watanabe K, Uno M, Yamashita S, Nakamura T, Masada M (2014). Usability assessment of laboratory data printed on the prescription for outpatients. IRYOUYAKUGAKU..

[7] Shikamura Y, Mano Y, Komoda M, Negishi K, Sato T, Miyazaki S (2016). Reduction of medical cost through pharmaceutical inquiries by community pharmacists and relation with Iyaku Bungyo rates: a nationwide survey on prescription inquiries. YAKUGAKU ZASSHI..

[8] Sakurai K, Ozaki J, Yano I, Adachi K, Kimura Y, Matsumura K, Nishiwaki F, Yoshida Y, Ikemi Y, Kayano Y, Yonezawa A, Fukatsu S, Matsubara K (2016). Protocol for unrequired inquiries about the prescription to doctors under the agreement between the hospital and community pharmacies. IRYOUYAKUGAKU..

[9] Kawazoe H, Ueno M, Sumikawa S, Tanaka M, Tanaka A, Araki H (2014). Evaluation of bidirectional sharing of patient information for S-1 between hospitals and community pharmacies using a modified prescription format. IRYOUYAKUGAKU..

[10] Kabeya M, Hibi S, Yuasa S, Inoue H, Saito A, Ina K (2015). Communication form of cooperation between hospitals and health insurance pharmacies designed based on the results of a questionnaire survey on cancer patients and community pharmacists. IRYOUYAKUGAKU..

[11] Sato Y, Morozumi I, Kawakami H, Suzuki M, Hasegawa Y, Okada C, Wakita A, Yamada K (2015). Survey on the use of chemotherapy labels and regimen worksheets to provision of information for community pharmacies. IRYOUYAKUGAKU..

[12] Oka A, Yonekawa Y, Suma K, Murata K (2012). A tool to share information about outpatient chemotherapy between hospital and pharmacy. Gan To Kagaku Ryoho..

[13] Ishibashi M, Ishii M, Nagano M, Kiuchi Y, Iwamoto S (2018). A questionnaire survey on cooperation between community pharmacies and hospitals in outpatient chemotherapy-comparison of roles of pharmacists in community pharmacy and hospitals. Yakugaku Zasshi..

[14] Choi Y, Nam J, Yang D, Jung W, Lee HR, Kim SH (2019). Effect of smartphone application-supported self-rehabilitation for frozen shoulder: a prospective randomized control study. Clin Rehabil..

[15] Wang HC, Chang FY, Tsai TM, Chen CH, Chen YY (2020). Development and clinical trial of a smartphone-based colorimetric detection system for self-monitoring of blood glucose. Biomed Opt Express..

[16] Moylan HB, Carrico CK, Lindauer SJ, Tüfekçi E (2019). Accuracy of a smartphone-based orthodontic treatment-monitoring application: a pilot study. Angle Orthod..

[17] Handa S, Okuyama H, Yamamoto H, Nakamura S, Kato Y (2020). Effectiveness of a smartphone application as a support tool for patients undergoing breast cancer chemotherapy: a randomized controlled trial. Clin Breast Cancer.

[18] Kim HJ, Kim SM, Shin H, Jang JS, Kim YI, Han DH (2018). A mobile game for patients with breast cancer for chemotherapy self-management and quality-of-life improvement: randomized controlled trial. J Med Internet Res..

[19] Cheong IY, An SY, Cha WC, Rha MY, Kim ST, Chang DK, Hwang JH (2018). Efficacy of mobile health care application and wearable device in improvement of physical performance in colorectal cancer patients undergoing chemotherapy. Clin Colorectal Cancer..

[20] Lyu KX, Zhao J, Wang B, Xiong GX, Yang WQ, Liu QH, Zhu XL, Sun W, Jiang AY, Wen WP, Lei WB (2016). Smartphone application WeChat for clinical follow-up of discharged patients with head and neck tumors: a randomized controlled trial. Chin Med J (Engl)..

[21] Pavic M, Klaas V, Theile G, Kraft J, Tröster G, Blum D, Guckenberger M (2020). Mobile health technologies for continuous monitoring of cancer patients in palliative care aiming to predict health status deterioration: a feasibility study. J Palliat Med..

[22] Urakawa R, Tarutani M, Kubota K, Uejima E (2019). Hand foot syndrome has the strongest impact on QOL in skin toxicities of chemotherapy. J Cancer..

